# N^6^-Methyladenosine regulator RBM15B acts as an independent prognostic biomarker and its clinical significance in uveal melanoma

**DOI:** 10.3389/fimmu.2022.918522

**Published:** 2022-08-08

**Authors:** Tianyu Wang, Jianhao Bai, Yuanyuan Zhang, Yawen Xue, Qing Peng

**Affiliations:** Department of Ophthalmology, Shanghai Tenth People’s Hospital of Tongji University, Tongji University School of Medicine, Shanghai, China

**Keywords:** m6A (N6-methyladenosine), RBM15B, uveal melanoma, prognosis, TCGA

## Abstract

Uveal melanoma (UM) is the most frequent intraocular malignant tumor in adults. N^6^-Methyladenosine (m^6^A) methylation is recognized as the most critical epigenetic change and is implicated in the development of many malignancies. However, its prognostic value in UM is poorly understood. RNA-seq and clinical data from The Cancer Genome Atlas (TCGA) help us better understand the relationship between m^6^A regulators and UM patients. Herein, four UM groups established by consensus clustering were shown to have different immune cell infiltrations and prognostic survival. Five m^6^A regulators, including RBM15B, IGF2BP1, IGF2BP2, YTHDF3, and YTHDF1, were associated with the prognosis of UM patients. Intriguingly, RBM15B was confirmed to be the only independent prognostic factor for UM and it was significantly correlated with clinicopathologic characteristics of UM. Notably, *RBM15B* expression was significantly negatively correlated with immune checkpoints. Furthermore, LINC00665/hsa-let-7b-5p/RBM15B axis and LINC00638/hsa-miR-103a-3p/RBM15B axis were found to be potential prognostic biomarkers in UM. In a nutshell, this work, through bioinformatics analysis, systematically described the gene signatures and prognostic values of m^6^A regulators. RBM15B is an independent protective prognostic factor, which may help us better understand the crosstalk within UM.

## Introduction

Uveal melanoma (UM) is the most frequent intraocular malignant tumor in adults, developing from melanocytes located in the choroid (90%), ciliary body (6%), or iris (4%) (1). UM is more common between the ages of 50 and 70, and it is quite uncommon among children (2). Although UM accounts for approximately 5% of all primary melanoma patients, nearly 50% of UM becomes metastatic and transfers to the liver, reducing the quality of life and adding a significant cost to people and society (3, 4). The emergence and progression of uveal malignant melanoma is a complex and multifactorial process. Several therapeutic approaches have been tried in UM, including enucleation, brachytherapy, stereotactic radiotherapy, and proton therapy; however, few hopeful outcomes have been reported (5, 6). Therefore, it is imperative to identify reliable biomarkers for prognostic prediction and targeted treatment.

m^6^A has recently garnered a lot of attention (7, 8). Discovered in 1974, m^6^A is described as the methylation of the nitrogen atom (N) at the sixth position of adenine (9). m^6^A is the most prevalent transcriptional modification of eukaryotic mRNA. This methylation modification of m^6^A is reversible, with the involvement of methyltransferases (writers), demethylases (erasers), and methyl-binding proteins (readers) (10, 11). Dysregulation of m^6^A modification has also been linked to tumorigenesis, prognosis, and treatment of various cancers (12–14). However, just a few research focused on its influence on UM (15–18). He and colleagues, for example, revealed that Beta-Secretase 2 (BACE2) presented an increased level of m6A RNA methylation, which led to the upregulation of BACE2 mRNA (16). A previous study revealed an association between ferroptosis-related lncRNAs and uveal melanoma and further identified a five genes novel signature which has effects on prognosis for UM patients (19). The m^6^A regulator METTL3 was markedly increased in UM cells and proved to be an important oncogenic factor in UM progression (18). However, the mechanism of the additional m^6^A regulators in uveal melanoma tumorigenesis warrants more investigation.

RNA Binding Motif Protein 15B (RBM15B), which regulates the alternative mRNA splicing and functions as an mRNA export factor, is essential for m^6^A methylation (20). RBM15B is a functional competitor of the serine-arginine protein, inhibiting the activity of the CDK11(p110)-cyclin L2α complex and acting as a new CDK11(p110) binding partner (21). The physiological and pathological role of *RBM15B* in uveal melanoma has been unknown so far.

As a result, we performed a systematic bioinformatics analysis to reveal the effect of m^6^A regulators on UM patients. For the first time, we demonstrated that RBM15B is an independent prognostic factor for UM. Furthermore, we built the LINC00665/hsa-let-7b-5p/RBM15B axis and LINC00638/hsa-miR-103a-3p/RBM15B axis to illustrate the function of RBM15B. These data indicate that RBM15B is a potential diagnostic and prognostic target for UM.

## Materials and methods

### Data acquisition and identification of m^6^A-related regulators

The TCGA database (https://portal.gdc.cancer.gov/) was used to gather all normalized RNA-seq and clinical data from UM patients (**Supplementary Table 1**). A total of 20 m^6^A-related regulators identified in the literature were included in the study, including seven writers (RBM15B, VIRMA, RBM15, METTL3, ZC3H13, WTAP, METTL14), two erasers (FTO, ALKBH5), and eleven readers (IGF2BP2, HNRNPA2B1, IGF2BP1, YTHDF3, IGF2BP3, HNRNPC, RBMX, YTHDC2, YTHDF1, YTHDC1, and YTHDF2) (**Supplementary Table 2**) (22).

### Genetic alteration and consensus clustering of m^6^A regulators

The cBioPortal (http://www.cbioportal.org/) database was used to determine the genetic changes in m^6^A regulators and their relationships to survival prognosis. The R software package “pheatmap” was used to show the 20 m^6^A regulators correlation map. Also, the geneMANIA online tool (https://genemania.org/) was used to examine the candidate proteins that were most connected with the 20 m^6^A regulators (23). The R package “consensus cluster plus” was used to divide the UM patients into four clusters to further understand the various etiology and clinical prognostic features of m^6^A. The maximum number of clusters was six, and in this procedure, 80% of the samples were drawn 100 times. The delta area curve of consensus clustering was then used to determine the relative change in area under the cumulative distribution function (CDF) curve. The R packages “ggplot2” and “pheatmap” were used to compare the expression distribution of m^6^A regulators across four groups.

### Differences in immune cell infiltration across four clusters

QUANTISEQ algorithm is based on a novel signature matrix and a constrained least square regression, which is specifically designed for RNA-seq analysis. It also performes well with deconvolution in different cancer types. In our study, it analyzed 10 immune cell types and uncharacterized cells. Meanwhile, MCPCOUNTER algorithm, another scoring method based on a stringent and robust set of marker genes, was also used in our study to quantify 8 immune cells, fibroblasts, and endothelial cells. The packages “survival” and “survminer” were used to construct the Kaplan-Meier ′ s survival curve of UM patients (24).

### Univariate and multivariate cox regression analyses

The “survival analysis” module from GEPIA2 (gene expression profiling interactive analysis 2) (http://gepia2.cancer-pku.cn) was used to perform survival analysis based on *RBM15B* expression (25), and the group cutoff was 50%. Furthermore, univariate and multivariate cox regression analyses were performed to investigate the potential correlation between 20 m^6^A regulators and OS of UM patients using the “survival” package (26). Furthermore, after demonstrating that RBM15B was the sole independent prognostic factor for OS, the R software packages “ggrisk”, “survival”, “survminer”, and “timeROC” were used to explore the survival value of RBM15B in UM patients.

### Enrichment and immune infiltration analyses

The low and high-RBM15B expression data were used in Gene Ontology (GO) and Kyoto Encyclopedia of Genes and Genomes (KEGG) studies to investigate signaling pathways activated in UM. The criteria were set as follows: |logFC|>2, p<0.01. The R packages “ggplot2” and “clusterprofiler” were used throughout this process (27). In addition, the TIMER2.0 (http://timer.cistrome.org/) online database was used to explore the potential correlation between the amount of immune cell infiltration and *RBM15B* expression based on the R packages “ggplot2” and “pheatmap” (24). Furthermore, the expression of eight immune checkpoints their correlation with *RBM15B* was also explored. The eight immune checkpoints include programmed death-ligand 1 (CD274), cytotoxic t-lymphocyte-associated protein 4 (CTLA4), hepatitis a virus cellular receptor 2 (HAVCR2), lymphocyte activating 3 (LAG3), programmed cell death 1 (PDCD1), programmed cell death 1 ligand 2 (PDCD1LG2), T cell immunoreceptor with Ig and ITIM domains (TIGIT), and sialic acid binding Ig like lectin 15 (SIGLEC15). The spearman correlation of tumor mutation burden (TMB) and *RBM15B* gene expression was also investigated (28, 29).

### Construction of ceRNA networks

Clinical data from the TCGA database were downloaded from the Genomic Data Commons (GDC) portal. ceRNA networks were established as follows: miRNAs and lncRNAs were predicted based on interactions in the Encyclopedia of RNA Interactomes (ENCORI); all predicted miRNAs and lncRNAs exhibited significant prognostic survival and correlations with RBM15B based on the OncomiR database.

All analyses were performed with the R software (version 4.0.3) and online databases (**Supplementary Table 3**).

## Results

### Different expressions of m^6^A regulators revealed by consensus clustering

**Figure 1:**
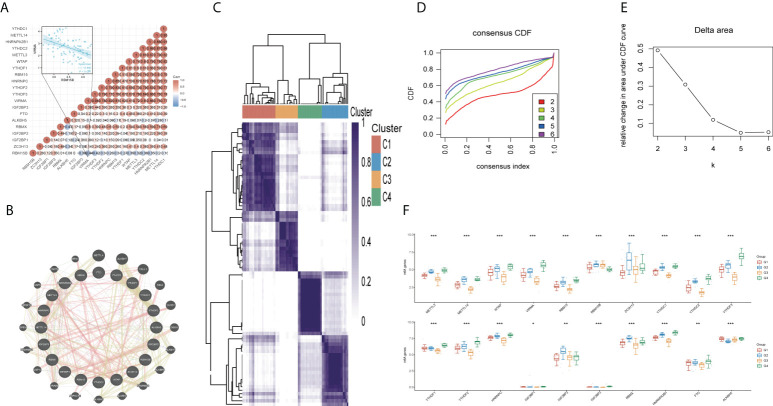
Correlations and consensus clustering analysis of 20 m^6^A regulators. **(A)** Heatmap of the correlation between the 20 m^6^A regulators. Red color represents positive correlation and blue represents negative correlation. **(B)** Twenty most associated molecules associated with 20 m^6^A regulators. **(C)** TCGA uveal melanoma cohort was classified into four clusters. **(D)** Cumulative distribution function (CDF) curve and **(E)** Delta area curve of consensus clustering. **(F)** Comparison of gene expression levels of 20 m^6^A regulators among four groups. *p<0.05, **p<0.01, ***p<0.001.

The analysis of genetic changes revealed the majority of m^6^A regulators had no genetic variants. As such, no significant correlation was found between m^6^A regulator mutations and overall survival (OS) or disease-free survival (DFS) (**Supplementary Figure 1**). According to the heatmap of the spearman correlation analysis, the 20 m^6^A regulators revealed complex relationships across writers, readers, and erasers. *RBM15B* was negatively correlated with most of the regulators, including *VIRMA* (**Figure 1A**, R = -0.440, *p* < 0.001). In addition, the GeneMANIA data indicated that the most associated molecules of m^6^A regulator were METTL4, ALKBH7, CBLL1, and RIDA in protein-protein interaction networks (**Figure 1B**). Consensus clustering analysis was performed in 80 UM patients based on the TCGA to better understand the association between the 20 m^6^A regulators and clinical prognosis features. The results showed that k=4 was the best value for stable clustering when k ranged between 1 and 6 (**Figures 1C-E**). The clinical characteristics of the four groups are illustrated in **Supplementary Figure 2**. The expression levels of these regulators were compared across the four groups; the findings revealed that all 20 m^6^A regulators were expressed differently. (all *p*<0.05) (**Figure 1F**).

### Immune cell infiltration contributes to different prognostic survivals

**Figure 2:**
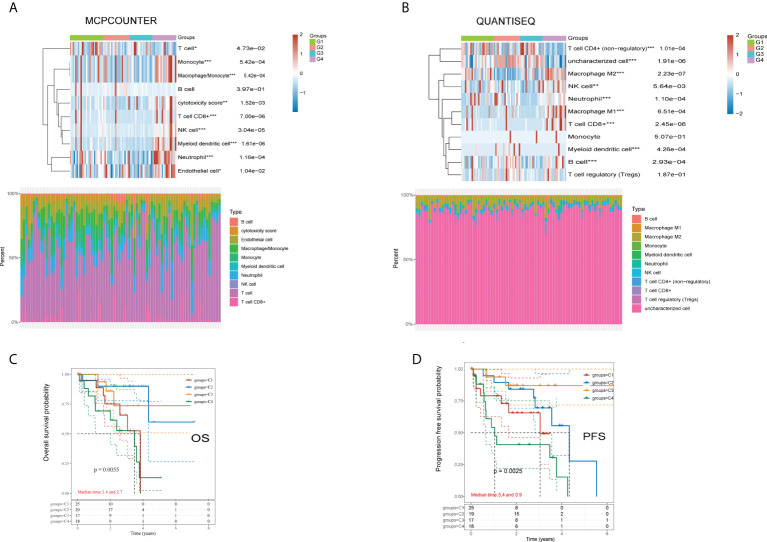
Differences of immune cells using QUANTISEQ and MCPCOUNTER algorithms, and overall survival and disease-free survival analysis in four groups. **(A)** MCPCOUNTER algorithms showed the differences of immune cells in different groups of samples. **(B)** QUANTISEQ algorithms showed the differences of immune cells in different groups of samples. **(C)** Kaplan-Meier OS survival analysis of the four different subtypes. **(D)** Kaplan-Meier PFS survival analysis of the four different subtypes. *p<0.05, **p<0.01, ***p<0.001.

There were significant disparities in immune cell infiltration among the four subgroups. Two algorithms (QUANTISEQ and MCPCOUNTER) were used to examine the variations in immune cells across the four groups. The MCPCOUNTER algorithm revealed a statistically significant difference among all immune cells except B cells (**Figure 2A**). At the same time, the QUANTISEQ algorithm demonstrated a statistically significant difference across all the immune cells except monocytes and regulatory T cells (**Figure 2B**). The analysis of the survival status of the four subgroups revealed that group 4 had a worse OS and PFS than the other 3 groups (**Figures 2C, D**).

### Key m^6^A regulators based on GEPIA and various cox regressions

**Figure 3:**
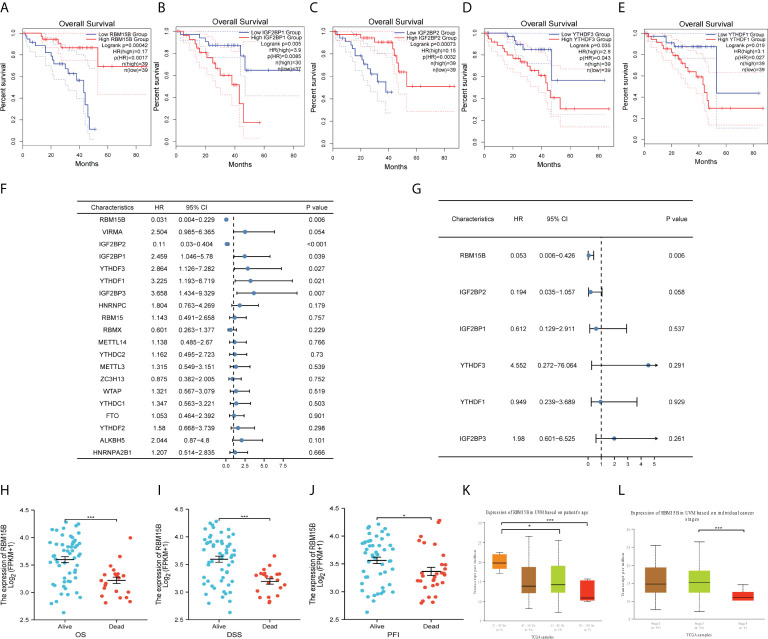
Identification of RBM15B as an independent prognostic biomarker and its correlation with clinical characteristics. Overall survivals of UM patients were calculated by Kaplan-Meier curves based on different expression of **(A)** *RBM15B*, **(B)** *IGF2BP1*, **(C)** *IGF2BP2*, **(D)** *YTHDF3* and **(E)** *YTHDF1*. **(F)** Univariate cox regression analysis and **(G)** Multivariate cox regression analysis of m^6^A regulators. *RBM15B* expression was significantly correlated with **(H)** OS, **(I)** DSS, **(J)** PFI, **(K)** Age and **(L)** Cancer stage. *p<0.05, ***p<0.001. OS, overall survival. DSS, disease specific survival. PFI, progression free interval.

Analysis of the GEPIA2 database revealed that five genes (*RBM15B, IGF2BP1, IGF2BP2, YTHDF3* and *YTHDF1*) significantly influenced the prognosis of UM patients (**Figures 3A-E**). Moreover, univariate and multivariate cox regression model were used to identify genes of prognostic significance. Univariate cox regression showed that RBM15B (*p*<0.001, HR=0.031), IGF2BP1 (*p*=0.039, HR=2.456), IGF2BP2 (*p*<0.001, HR=0.110), IGF2BP3 (*p*=0.007, HR=3.658), YTHDF3 (*p*=0.027, HR=2.864), and YTHDF1 (*p*=0.021, HR=3.225) were correlated with UM prognosis (**Figure 3F**). Multivariate cox regression demonstrated that RBM15B was the sole independent prognostic factor for OS (*p*=0.006, HR=0.053) (**Figure 3G**, **Supplementary Table 4**).

### Correlation of RBM15B expression with clinicopathologic features

*RBM15B* was discovered in the nucleoplasm and its RNA expression exhibited a low tissue specificity; however, it was not translated into proteins in certain tissues (**Supplementary Figure 3**). *RBM15B* had no genetic variants, according to genomic analysis. As such, no significant correlation was revealed between RBM15B and OS abnormalities and disease-specific survival (DSS) or progression-free survival (PFS) (**Supplementary Figure 4**). We separated UM patients into distinct subgroups to reveal the correlations between RBM15B and clinicopathologic features. The results demonstrated that increased levels of *RBM15B* expression were significantly correlated with improved OS, DSS, and progression-free interval (PFI) (**Figures 3H-J**). Moreover, significantly different *RBM15B* expression was observed in subgroups of patients’ age and cancer stages based on the UALCAN database (**Figures 3K, L**) (30). Logistic regression analysis revealed a significant correlation of *RBM15B* expression with clinical stage and histological type (*p*<0.05) (**Supplementary Table 5**).

### Predictive power of RBM15B expression and GO and KEGG enrichment analyses in UM

**Figure 4:**
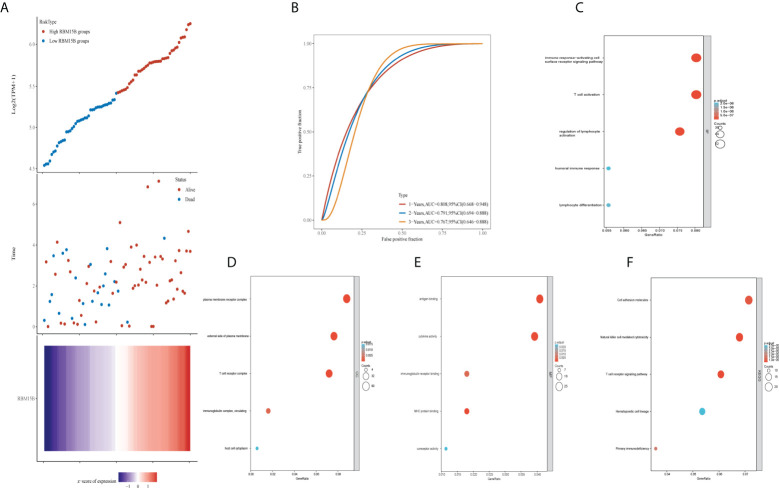
Prognostic value and GO and KEGG analysis of RBM15B. **(A)** Patients were divided into low and high groups based on *RBM15B* expression. **(B)** Time-dependent receiver operating characteristic analysis of RBM15B. Bubble charts showed the top 5 elements of **(C)** Biological process; **(D)** Cellular component; **(E)** Molecular function; and **(F)** Kyoto encyclopedia of genes and genomes analysis. GO, gene oncology, KEGG, kyoto encyclopedia of genes and genomes.

The scatter plot, heat map of gene expression, and receiver operating characteristic curves were used to evaluate the prognostic and diagnostic significance of *RBM15B* expression. The dotted line separated patients into low-risk and high-risk groups. According to the scatter plot and gene expression heatmap, UM patients with a higher *RBM15B* expression had a better OS than those with a low level of *RBM15B* expression (*p*<0.001) (**Figure 4A**). The time-dependent ROC analysis demonstrated that the area under the curve (AUC) for different survival years was 0.808, 0.791, and 0.767, respectively (**Figure 4B**). In addition, GO analysis revealed that *RBM15B* expression was primarily associated with immune-related gene terms: BP terms, including immune response-activating cell surface receptor signaling pathway, T cell activation, regulation of lymphocyte activation, humoral immune response, and lymphocyte differentiation (**Figure 4C**); CC terms, including plasma membrane receptor complex, external side of the plasma membrane, T cell receptor complex, immunoglobulin complex circulating, and host cell cytoplasm (**Figure 4D**); MF terms, including antigen-binding, cytokine activity, immunoglobulin receptor binding, MHC protein binding, and coreceptor activity (**Figure 4E**). KEGG pathway analysis revealed that *RBM15B* was significantly associated with natural killer cell-mediated cytotoxicity, T cell receptor signaling pathways, and primary immunodeficiency pathways (**Figure 4F**).

### Correlations between RBM15B expression and immune checkpoints

**Figure 5:**
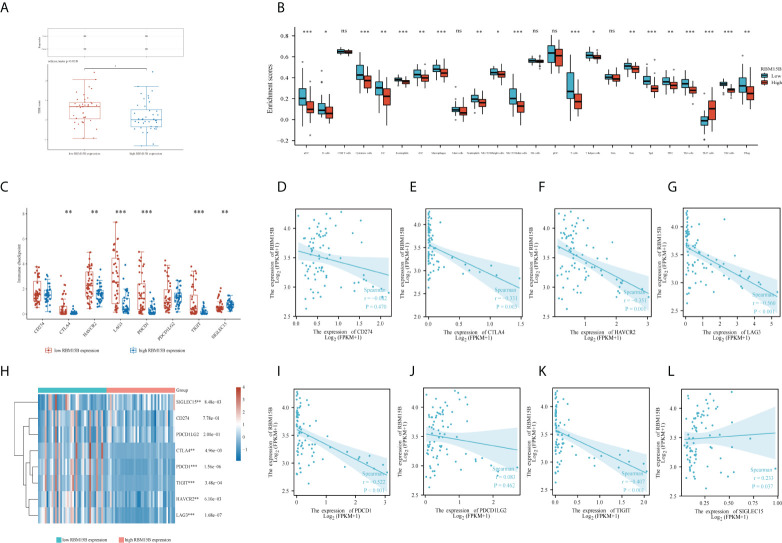
Function and pathway enrichment analysis of RBM15B and CD8(+) immune infiltrations in uveal melanoma cases. **(A)** distribution of immune response scores between high and low *RBM15B* expression groups in uveal melanoma. **(B)** Immune cell infiltration between low and high expression of *RBM15B* groups. **(C)** Expression distribution of immune checkpoints gene between low and high expression of *RBM15B* groups. Spearman correlation of RBM15B with expression of **(D)** CD274; **(E)** CTLA4; **(F)** HAVCR2; **(G)** LAG3 in UM. **(H)** Heatmap of immune checkpoints gene expression. The different colors represent the trend of gene expression in different samples. Spearman correlation of RBM15B with expression of **(I)** PDCD1; **(J)** PDCD1LG2; **(K)** TIGIT; **(L)** SIGLEC15 in UM. *p<0.05, **p<0.01, ***p<0.001. ns, no significance.

The tumor immune dysfunction and exclusion (TIDE) algorithm was used to predict the potential immune checkpoint blockade (ICB) response (31). This demonstrated the significant difference in immune response scores between high and low *RBM15B* expression groups (**Figure 5A**). Then, using the R package “GSVA”, we analyzed the enrichment score distribution of immune cells between low and high *RBM15B* expression groups (**Figure 5B**); the results revealed significant differences in most of immune cells, except for CD 8+ T cell, mast cells, natural killer (NK) cells, plasmacytoid dendritic cell (pDC), and Central Memory T cells (32, 33). However, no significant relationships between *RBM15B* expression and tumor mutation burden (TMB) score or microsatellite instability (MSI) scores were observed (**Supplementary Figure 5**).

Additionally, the expression of eight immune checkpoints (including CD274, CTLA4, HAVCR2, LAG3, PDCD1, PDCD1LG2, TIGIT, and SIGLEC15) was compared between patients with low and high *RBM15B* expression. The results showed that patients with low *RBM15B* expression expressed more immune checkpoints (except for CD274 and PDCD1LG2) (**Figures 5C, H**). Furthermore, RBM15B was significantly negatively correlated with the expression of most of immune checkpoints (except for CD274 and PDCD1LG2) (**Figures 5D-G, I-L**).

### Construction of networks of LncRNA-miRNA-mRNA

**Figure 6:**
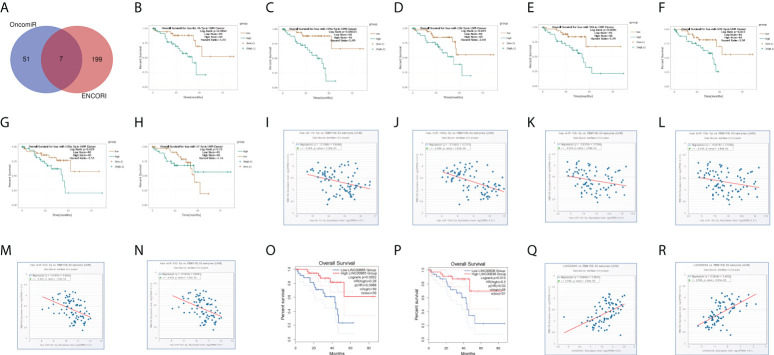
competing endogenous RNA network construction. **(A)** 7 potential target miRNAs were obtained from ENCORI and OncomiR databases. **(B-H)** Kaplan-Meier plots revealed the overall survival of 7 potential target miRNAs, including **(B)** hsa-let-7b-5p, **(C)** hsa-miR-199a-5p, **(D)** hsa-miR-10b-5p, **(E)** hsa-miR-346, **(F)** hsa-miR-532-5p, **(G)** hsa-miR-103a-3p and **(H)** hsa-miR-17-5p. **(I–N)** among 7 potential target miRNAs, 6 miRNAs, **(I)** hsa-let-7b-5p, **(J)** hsa-miR-199a-5p, **(K)** hsa-miR-10b-5p, **(L)** hsa-miR-346, **(M)** hsa-miR-532-5p, and **(N)** hsa-miR-103a-3p significantly correlated the expression of *RBM15B*. Kaplan-Meier plots revealed the overall survival of 2 potential target lncRNAs, **(O)** LINC00665 and **(P)** LINC00638, predicted by ENCORI. 2 potential target lncRNAs, **(Q)** LINC00665 and **(R)** LINC00638, significantly correlated the expression of *RBM15B*.

ENCORI and OncomiR databases were used to filter out 51 and 199 candidate miRNAs, respectively, to further investigate the potential functions of *RBM15B*. These candidate target miRNAs were then intersected to yield seven miRNAs (**Figure 6A**), of which hsa-let-7b-5p, hsa-miR-199a-5p, hsa-miR-10b-5p, hsa-miR-346, hsa-miR-532-5p, and hsa-miR-103a-3p were identified as potential *RBM15B* targets; this was based on their prognostic values and correlations with *RBM15B*. All these six miRNAs were strongly linked with *RBM15B* expression. Also, increased expression of these six miRNAs had an adverse prognostic value for UM patients (**Figures 6B-N**). The ENCORI database was used to identify potential lncRNAs, and the results revealed LINC00665 and LINC00638 as the potential target based on their expression levels and associations with hsa-let-7b-5p, hsa-miR-103a-3p, and *RBM15B* (**Figures 6O-R**). In this view, the LINC00665/hsa-let-7b-5p/RBM15B axis and LINC00638/hsa-miR-103a-3p/RBM15B axis were constructed, which are potential prognostic biomarkers in UM.

## Discussion

Uveal melanoma is a cancerous condition that poses a threat to people’s health, and UM is different from cutaneous melanoma in terms of genetic background and clinical behavior (34). Therefore, finding effective treatment approaches for this cancer is critical. Subgroup classification is crucial in uveal melanoma because it dictates the specificity of treatment and prognosis (35, 36). The present investigation using m^6^A-related regulators classified TCGA patients into 4 groups and narrowed them down to the relevant subgroups for different analysis. The analysis revealed cross-talk among 20 m^6^A regulators in UM patients, suggesting that m^6^A may play important roles in the course and prognosis of UM cases. Consensus clustering analysis suggested that different expression levels of 20 m^6^A regulators resulted in different survival prognoses among four UM groups, indicating that the 20 m^6^A regulators have a potentially significant impact on the prognosis of UM patients. Moreover, two algorithms (QUANTISEQ and MCPCOUNTER) revealed multiple statistically significant changes in immune cell populations across the four UM groups. Intriguingly, CD8(+) T cells, myeloid dendritic cells, and macrophages were the most statistically significant infiltrating cells. we found that different m^6^A regulator expressions in these four subgroups contributed to different prognosis, but these four subgroups also had different immune cell infiltrations. Thus, we had the hypothesis that m^6^A regulators may correlate with immune cells to influence patients’ prognosis.

However, it was unclear which of the m^6^A regulators might impact the survival prognosis of UM patients. *RBM15B, IGF2BP2, IGF2BP1, YTHDF3*, and *YTHDF1* with significantly results were identified as candidate genes in the GEPIA2 database. Meanwhile, univariate and multivariate cox regression analyses revealed that 6 regulators (including RBM15B, IGF2BP1, IGF2BP2, IGF2BP3, YTHDF3, and YTHDF1) were associated with UM prognosis, and RBM15B was the only independent prognostic factor for OS. These results were quite encouraging; therefore, we focused on the molecular characterization and possible clinical applications of the *RBM15B* gene.

RBM15B is one of the most essential N^6^-Methyladenosine methyltransferases. Emerging evidence implicates RBM15B plays an important part in carcinoma growth and metastasis in several cancers (37, 38). Zhang and colleagues, for example, demonstrated that *RBM15B* was highly expressed in ovarian cancer and increased expression of *RBM15B* correlated with worse PFS and ovarian cancer cell metastasis (37). In addition, another study showed that *RBM15B* was highly expressed in hepatocellular carcinoma, and it promoted hepatocellular carcinoma cell growth, invasion and metastasis *in vivo* and *in vitro*, thus resulting in a poor prognosis (38). In our study, Logistic regression analysis revealed a significant correlation of RBM15B expression with clinical stage and histological type. Moreover, stage 4 of uveal melanoma had a markedly lower expression level compared with stage 3, indicating that RBM15B inhibit tumor growth and progression. Our present investigation also revealed that increased *RBM15B* levels were strongly linked with improved OS, DSS, and PFI in UM patients. Therefore, we confirmed that RBM15B inhibit UM growth and progression. We also speculate that RBM15B inhibits the metastasis of UM patients.

Furthermore, multivariate Cox regression revealed that RBM15B was the only independent prognostic factor for UM patients. But what exactly is the mechanism? Interestingly, GO and KEGG enrichment analysis confirmed that *RBM15B* expression was primarily associated with immune-related terms (including T cell activation, T cell receptor complex, and humoral immune response) and pathways (including T cell receptor signaling pathway), implying that RBM15B may influence the survival prognosis of UM patients by regulating specific immune-related pathways. In this view, we looked at the relationships between *RBM15B* expression and several immune checkpoint molecules. Immune checkpoints are expressed on a wide range of immune cells, which suppresses the immune function. When immune cells fail to mount an effective anti-tumor immune response, tumor immune escape causes tumor progression and distant metastasis. Our study revealed that *RBM15B* expression was significantly negatively correlated with 6 immune checkpoints (including CTLA4, HAVCR2, LAG3, PDCD1, TIGIT, and SIGLEC15). When *RBM15B* is highly expressed, 6 immune checkpoints are low expressed. Thus, we believe that there is a reciprocal association between *RBM15B* and 6 immune checkpoints expressions. *RBM15B* may affect the expressions of 6 immune checkpoints, or 6 immune checkpoints may affect the expressions of *RBM15B*, or both *RBM15B* and 6 immune checkpoints are regulated by a third unknown factor in organism. The question which is the right one needs verification in our next work. Briefly speaking, *RBM15B* expression was negatively correlated with 6 immune checkpoints, thus influencing the prognosis of UM patients.

The ceRNA regulation of RBM15B, including long noncoding RNAs (lncRNAs) and microRNAs, was also investigated in this work. It has been reported mRNAs and lncRNAs “talk” to each other using microRNA response elements (MREs) as letters of a new language. MicroRNAs can decrease the stability of target RNAs and prohibit their translation. Seven predicted programs (including microT, miRanda, miRmap, PITA, RNA22, PicTar, TargetScan) were used to predict miRNA-RBM15B interactions to identify the upstream miRNAs of RBM15B. Meanwhile, the OncomiR database was used to extract a list of miRNAs that are strongly linked to UM patient survival. The results showed 7 miRNAs that functioned as oncogenic miRNAs and showed markedly negative correlations with RBM15B. has-let-7b-5p, for example, exhibited a negative correlation with RBM15B (r=-0.309, p=5.26e-03) and had an unfavorable overall survival in UM (p=0.0064). Similarly, according to the ceRNA theory, we discovered 2 potential lncRNAs, LINC00665 and LINC00638 (39). Survival analysis and correlation analysis demonstrated that LINC00665/hsa-let-7b-5p/RBM15B and LINC00638/hsa-miR-103a-3p/RBM15B axes are prognostic biomarkers in UM.

Some limitations should be noted when drawing conclusions from our study. Firstly, since our results are generated from bioinformatics analysis, one severe limitation is that no validation experiments on human samples were performed. Secondly, the data used in this study was downloaded from TCGA and other online databases, more clinical data and experiments are needed to further confirm the prognostic value of RBM15B in UM.

**Figure 7:**
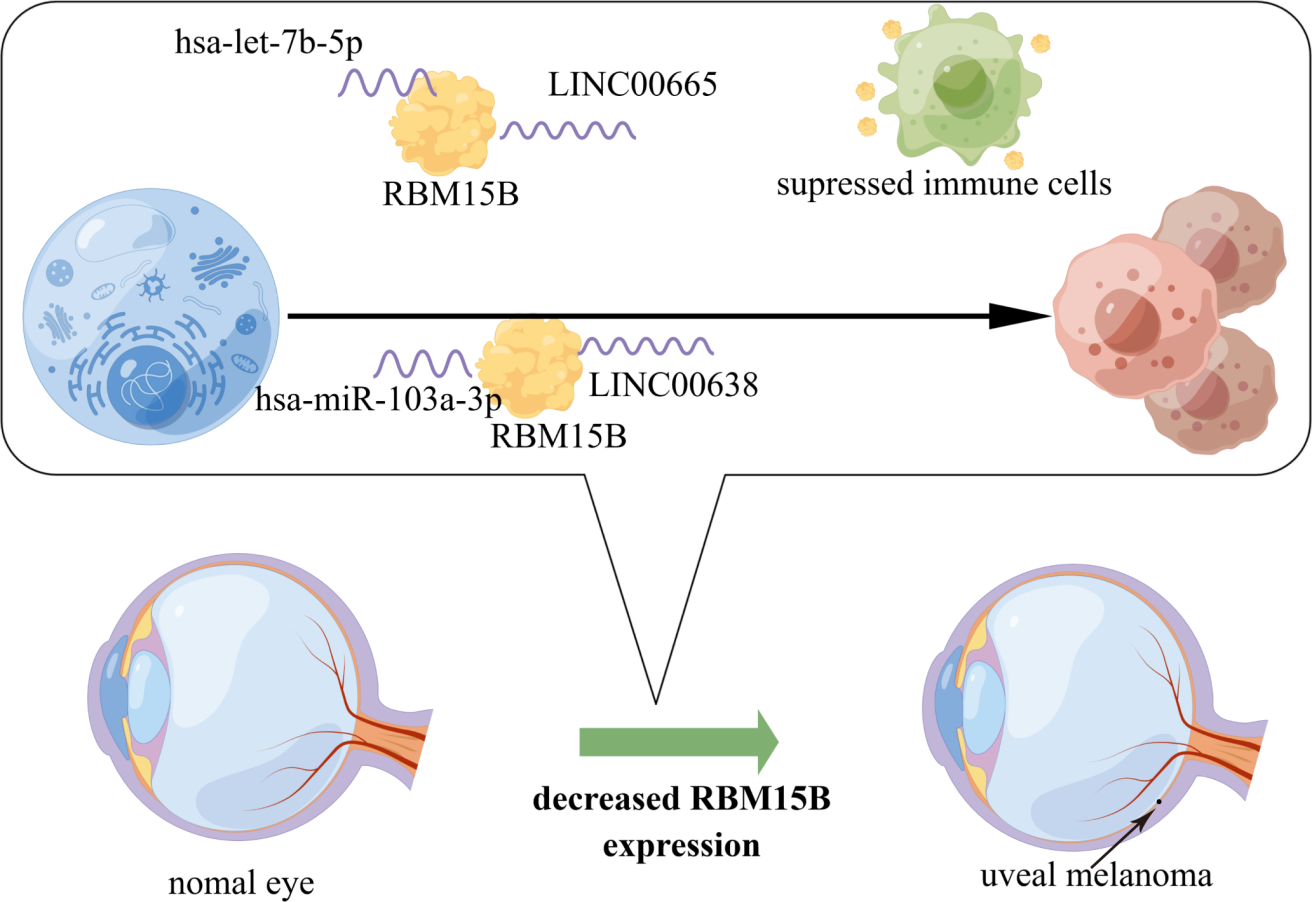
The model of regulatory mechanism of *RBM15B* in carcinogenesis of UM.

In conclusion, this study investigated 20 m^6^A-related regulators, performed m^6^A regulator consensus clustering, and found that RBM15B was the only independent prognostic factor for UM. Two upstream ceRNA regulation mechanisms of RBM15B were also identified in UM. In addition, it also showed that RBM15B positively influenced the survival prognosis of UM patients by decreasing the expression of immune checkpoints (**Figure 7**). However, further in-depth molecular research and large clinical trials would be necessary for the future to verify these findings.

## Data availability statement

The original contributions presented in the study are included in the article/**Supplementary Material**. Further inquiries can be directed to the corresponding author.

## Ethics statement

This study was exempted from approval by the institutional ethics committee of Shanghai Tenth People’s Hospital affiliated to Tongji University School of Medicine, China, and the need for informed consent was also waived for its data were obtained from publicly available databases that have approvals.

## Author contributions

TW: Writing- Original draft preparation, Conceptualization, Methodology, Data curation, Validation, Formal analysis. JB: Visualization, Investigation, Formal analysis, Resources. YZ and YX: Conceptualization, Methodology. Software, Validation. QP: Supervision, funding acquisition, Project administration. All authors contributed to the article and approved the submitted version.

## Fundings

This work was supported by the National Natural Science Foundation of China [grant number 81470029]; the Shanghai Municipal Health Bureau [grant number ZY (2018-2020)-ZWB-1001-CPJS10]; and the Three-Year Action Plan for Promoting Clinical Skills and Clinical Innovation in Municipal Hospitals [grant number SHDC2020CR5014].

## Acknowledgments

The authors thank the all the contributors of the TCGA (https://tcga-data.nci.nih.gov/) database and the other databases and concerned authors for sharing their data on open access. The authors also thank Figdraw (www.Figdraw.com) website.

## Conflict of interest

The authors declare that the research was conducted in the absence of any commercial or financial relationships that could be construed as a potential conflict of interest.

## Publisher’s note

All claims expressed in this article are solely those of the authors and do not necessarily represent those of their affiliated organizations, or those of the publisher, the editors and the reviewers. Any product that may be evaluated in this article, or claim that may be made by its manufacturer, is not guaranteed or endorsed by the publisher.

## Supplementary material

Supplementary Figure 1 overall survival and disease-free survival of patients between the aberrations of m^6^A regulators. **(A)** Kaplan-Meier plot revealed the overall survival of patients between the altered aberration group and the unaltered aberration group of m^6^A regulators. **(B)** Kaplan-Meier plot revealed the disease-free survival of patients between the altered aberration group and the unaltered aberration group of m^6^A regulators.

Supplementary Figure 2 Clinical characteristics of four groups from 80 UM patients based on TCGA database.

Supplementary Figure 3 Location of RBM15B and the expression and protein level of RBM15B.

Supplementary Figure 4 Alteration frequency of RBM15B and its influence on prognostic survivals. **(A)** Alteration frequency of RBM15B across pan-cancers from TCGA database. **(B)** Kaplan-Meier plot revealed the overall survival of patients between the altered aberration group and the unaltered aberration group of RBM15B. **(C)** Kaplan-Meier plot revealed the disease specific survival of patients between the altered aberration group and the unaltered aberration group of RBM15B. **(D)** Kaplan-Meier plot revealed the progression free survival of patients between the altered aberration group and the unaltered aberration group of RBM15B.

Supplementary Figure 5 correlations between *RBM15B* expression and TMB score and between *RBM15B* expression and MSI score. **(A)** Correlation between *RBM15B* expression and MSI score across pan-cancers. **(B)** Correlation between *RBM15B* expression and TMB score across pan-cancers. **(C)** Correlation between *RBM15B* expression and MSI score in UM. **(D)** Correlation between *RBM15B* expression and TMB score in UM. Abbreviations: TMB: tumor mutation burden; MSI: microsatellite instability

Supplementary Table 1 The clinical characteristics of the uveal melanoma patients based on TCGA.

Supplementary Table 2 The descriptions of 20 m^6^A regulators described in this study.

Supplementary Table 3 online database used in this study.

Supplementary Table 4 Univariate and multivariate cox analysis of 20 m^6^A regulators in uveal melanoma cases.

Supplementary Table 5 Logistic regression analysis of the correlation between RBM15B expression and clinical characteristics.

## References

[1] Kaliki S Shields CL. Uveal melanoma: Relatively rare but deadly cancer. Eye (Lond) (2017) 31(2):241–57. doi: 10.1038/eye.2016.275 PMC5306463 27911450

[2] Al-Jamal RT Cassoux N Desjardins L Damato B Konstantinidis L Coupland SE. The pediatric choroidal and ciliary body melanoma study: A survey by the European ophthalmic oncology group. Ophthalmology (2016) 123(4):898–907. doi: 10.1016/j.ophtha.2015.12.024 26854035

[3] Jager MJ Shields CL Cebulla CM Abdel-Rahman MH Grossniklaus HE Stern MH. Uveal melanoma. Nat Rev Dis Primers (2020) 6(1):24. doi: 10.1038/s41572-020-0158-0 32273508

[4] Gallenga CE Franco E Adamo GG Violanti SS Tassinari P Tognon M. Genetic basis and molecular mechanisms of uveal melanoma metastasis: A focus on prognosis. Front Oncol (2022) 12:828112. doi: 10.3389/fonc.2022.828112 35480119 PMC9037634

[5] Damato B. Ocular treatment of choroidal melanoma in relation to the prevention of metastatic death - a personal view. Prog Retinal Eye Res (2018) 66:187–99. doi: 10.1016/j.preteyeres.2018.03.004 29571968

[6] Fagone P Caltabiano R Russo A Lupo G Anfuso CD Basile MS. Identification of novel chemotherapeutic strategies for metastatic uveal melanoma. Sci Rep (2017) 7:44564. doi: 10.1038/srep44564 28303962 PMC5355998

[7] He PC He C. M(6) a rna methylation: From mechanisms to therapeutic potential. EMBO J (2021) 40(3):e105977. doi: 10.15252/embj.2020105977 33470439 PMC7849164

[8] Nombela P Miguel-Lopez B Blanco S. The role of M(6)a, M(5)C and psi rna modifications in cancer: Novel therapeutic opportunities. Mol Cancer (2021) 20(1):18. doi: 10.1186/s12943-020-01263-w 33461542 PMC7812662

[9] Desrosiers R Friderici K Rottman F. Identification of methylated nucleosides in messenger rna from novikoff hepatoma cells. Proc Natl Acad Sci USA (1974) 71(10):3971–5. doi: 10.1073/pnas.71.10.3971 PMC434308 4372599

[10] Bi Z Liu Y Zhao Y Yao Y Wu R Liu Q. A dynamic reversible rna N(6) -methyladenosine modification: Current status and perspectives. J Cell Physiol (2019) 234(6):7948–56. doi: 10.1002/jcp.28014 30644095

[11] Yang Y Hsu PJ Chen YS Yang YG. Dynamic transcriptomic M(6)a decoration: Writers, erasers, readers and functions in rna metabolism. Cell Res (2018) 28(6):616–24. doi: 10.1038/s41422-018-0040-8 PMC5993786 29789545

[12] Huang H Weng H Chen J. M(6)a modification in coding and non-coding rnas: Roles and therapeutic implications in cancer. Cancer Cell (2020) 37(3):270–88. doi: 10.1016/j.ccell.2020.02.004 PMC7141420 32183948

[13] He L Li H Wu A Peng Y Shu G Yin G. Functions of N6-methyladenosine and its role in cancer. Mol Cancer (2019) 18(1):176. doi: 10.1186/s12943-019-1109-9 31801551 PMC6892141

[14] Shen S Zhang R Jiang Y Li Y Lin L Liu Z. Comprehensive analyses of M6a regulators and interactive coding and non-coding rnas across 32 cancer types. Mol Cancer (2021) 20(1):67. doi: 10.1186/s12943-021-01362-2 33849552 PMC8045265

[15] Jia R Chai P Wang S Sun B Xu Y Yang Y. M(6)a modification suppresses ocular melanoma through modulating Hint2 mrna translation. Mol Cancer (2019) 18(1):161. doi: 10.1186/s12943-019-1088-x 31722709 PMC6854757

[16] He FL Yu J Yang J Wang SY Zhuang A Shi HH. M(6)a rna hypermethylation-induced Bace2 boosts intracellular calcium release and accelerates of ocular melanoma. Mol Ther (2021) 29(6):2121–33. doi: 10.1016/j.ymthe.2021.02.014 PMC8178445 33601055

[17] Tang J Wan Q Lu J. The prognostic values of M6a rna methylation regulators in uveal melanoma. BMC Cancer (2020) 20(1):674. doi: 10.1186/s12885-020-07159-8 32682400 PMC7368742

[18] Luo G Xu W Zhao Y Jin S Wang S Liu Q. Rna M(6) a methylation regulates uveal melanoma cell proliferation, migration, and invasion by targeting c-met. J Cell Physiol (2020) 235(10):7107–19. doi: 10.1002/jcp.29608 32017066

[19] Ma X Yu S Zhao B Bai W Cui Y Ni J. Development and validation of a novel ferroptosis-related lncrna signature for predicting prognosis and the immune landscape features in uveal melanoma. Front Immunol (2022) 13:922315. doi: 10.3389/fimmu.2022.922315 35774794 PMC9238413

[20] Patil DP Chen CK Pickering BF Chow A Jackson C Guttman M. M(6)a rna methylation promotes xist-mediated transcriptional repression. Nature (2016) 537(7620):369–73. doi: 10.1038/nature19342 PMC5509218 27602518

[21] Loyer P Busson A Trembley JH Hyle J Grenet J Zhao W. The rna binding motif protein 15b (Rbm15b/Ott3) is a functional competitor of serine-arginine (Sr) proteins and antagonizes the positive effect of the Cdk11p110-cyclin L2alpha complex on splicing. J Biol Chem (2011) 286(1):147–59. doi: 10.1074/jbc.M110.192518 PMC3012969 21044963

[22] Li Y Xiao J Bai J Tian Y Qu Y Chen X. Molecular characterization and clinical relevance of M(6)a regulators across 33 cancer types. Mol Cancer (2019) 18(1):137. doi: 10.1186/s12943-019-1066-3 31521193 PMC6744659

[23] Warde-Farley D Donaldson SL Comes O Zuberi K Badrawi R Chao P. The genemania prediction server: Biological network integration for gene prioritization and predicting gene function. Nucleic Acids Res (2010) 38(Web Server issue):W214–20. doi: 10.1093/nar/gkq537 PMC2896186 20576703

[24] Li T Fu J Zeng Z Cohen D Li J Chen Q. Timer2.0 for analysis of tumor-infiltrating immune cells. Nucleic Acids Res (2020) 48(W1):W509–W14. doi: 10.1093/nar/gkaa407 PMC7319575 32442275

[25] Tang Z Li C Kang B Gao G Li C Zhang Z. Gepia: A web server for cancer and normal gene expression profiling and interactive analyses. Nucleic Acids Res (2017) 45(W1):W98–102. doi: 10.1093/nar/gkx247 28407145 PMC5570223

[26] Liu J Lichtenberg T Hoadley KA Poisson LM Lazar AJ Cherniack AD. An integrated tcga pan-cancer clinical data resource to drive high-quality survival outcome analytics. Cell (2018) 173(2):400–16.e11. doi: 10.1016/j.cell.2018.02.052 29625055 PMC6066282

[27] Yu G Wang LG Han Y He QY. Clusterprofiler: An r package for comparing biological themes among gene clusters. OMICS (2012) 16(5):284–7. doi: 10.1089/omi.2011.0118 PMC3339379 22455463

[28] Thorsson V Gibbs DL Brown SD Wolf D Bortone DS Ou Yang TH. The immune landscape of cancer. Immunity (2018) 48(4):812–30 e14. doi: 10.1016/j.immuni.2018.03.023 29628290 PMC5982584

[29] Bonneville R Krook MA Kautto EA Miya J Wing MR Chen HZ. Landscape of microsatellite instability across 39 cancer types. JCO Precis Oncol (2017) 2017. doi: 10.1200/PO.17.00073 PMC5972025 29850653

[30] Chandrashekar DS Bashel B Balasubramanya SAH Creighton CJ Ponce-Rodriguez I Chakravarthi B. Ualcan: A portal for facilitating tumor subgroup gene expression and survival analyses. Neoplasia (2017) 19(8):649–58. doi: 10.1016/j.neo.2017.05.002 PMC5516091 28732212

[31] Jiang P Gu S Pan D Fu J Sahu A Hu X. Signatures of T cell dysfunction and exclusion predict cancer immunotherapy response. Nat Med (2018) 24(10):1550–8. doi: 10.1038/s41591-018-0136-1 PMC6487502 30127393

[32] Bindea G Mlecnik B Tosolini M Kirilovsky A Waldner M Obenauf AC. Spatiotemporal dynamics of intratumoral immune cells reveal the immune landscape in human cancer. Immunity (2013) 39(4):782–95. doi: 10.1016/j.immuni.2013.10.003 24138885

[33] Hanzelmann S Castelo R Guinney J. Gsva: Gene set variation analysis for microarray and rna-seq data. BMC Bioinf (2013) 14:7. doi: 10.1186/1471-2105-14-7 PMC3618321 23323831

[34] Russo D Di Crescenzo RM Broggi G Merolla F Martino F Varricchio S. Expression of P16ink4a in uveal melanoma: New perspectives. Front Oncol (2020) 10:562074. doi: 10.3389/fonc.2020.562074 33154942 PMC7590828

[35] Fallico M Raciti G Longo A Reibaldi M Bonfiglio V Russo A. Current molecular and clinical insights into uveal melanoma (Review). Int J Oncol (2021) 58(4). doi: 10.3892/ijo.2021.5190 PMC7910016 33649778

[36] Branisteanu DC Bogdanici CM Branisteanu DE Maranduca MA Zemba M Balta F. Uveal melanoma diagnosis and current treatment options (Review). Exp Ther Med (2021) 22(6):1428. doi: 10.3892/etm.2021.10863 34707709 PMC8543295

[37] Zhang L Luo M Yang H Zhu S Cheng X Qing C. Next-generation sequencing-based genomic profiling analysis reveals novel mutations for clinical diagnosis in Chinese primary epithelial ovarian cancer patients. J Ovarian Res (2019) 12(1):19. doi: 10.1186/s13048-019-0494-4 30786925 PMC6381667

[38] Tan C Xia P Zhang H Xu K Liu P Guo D. Yy1-targeted Rbm15b promotes hepatocellular carcinoma cell proliferation and sorafenib resistance by promoting Tram2 expression in an M6a-dependent manner. Front Oncol (2022) 12:873020. doi: 10.3389/fonc.2022.873020 35494016 PMC9046568

[39] Salmena L Poliseno L Tay Y Kats L Pandolfi PP. A cerna hypothesis: The Rosetta stone of a hidden rna language? Cell (2011) 146(3):353–8. doi: 10.1016/j.cell.2011.07.014 PMC3235919 21802130

