# Supplementary material for: N6-Methyladenosine regulator RBM15B acts as an independent prognostic biomarker and its clinical significance in uveal melanoma
Source: Front Immunol. 2022 Aug 8;13:918522. doi: 10.3389/fimmu.2022.918522 (PMC9393712; doi:10.3389/fimmu.2022.918522)
Supplement: Supplementary Table 1 — The clinical characteristics of the uveal melanoma patients based on TCGA. [file Table_1.docx]

**Table S1.** The clinical characteristics of the uveal melanoma patients based on TCGA.

| Characteristic | levels | Overall |
| --- | --- | --- |
| n |  | 80 |
| Pathologic T stage, n (%) | T2 | 14 (17.5%) |
|  | T3 | 32 (40%) |
|  | T4 | 34 (42.5%) |
| Pathologic N stage, n (%) | N0 | 52 (65.8%) |
|  | NX | 27 (34.2%) |
| Pathologic M stage, n (%) | M0 | 51 (65.4%) |
|  | M1 | 4 (5.1%) |
|  | MX | 23 (29.5%) |
| Pathologic stage, n (%) | Stage II | 39 (49.4%) |
|  | Stage III | 36 (45.6%) |
|  | Stage IV | 4 (5.1%) |
| Clinical T stage, n (%) | T2 | 4 (5.1%) |
|  | T3 | 36 (46.2%) |
|  | T4 | 38 (48.7%) |
| Clinical N stage, n (%) | N0 | 76 (95%) |
|  | NX | 4 (5%) |
| Clinical M stage, n (%) | M0 | 73 (91.2%) |
|  | M1 | 3 (3.8%) |
|  | MX | 4 (5%) |
| Clinical stage, n (%) | Stage II | 36 (45%) |
|  | Stage III | 40 (50%) |
|  | Stage IV | 4 (5%) |
| Gender, n (%) | Female | 35 (43.8%) |
|  | Male | 45 (56.2%) |
| Age, n (%) | <=60 | 40 (50%) |
|  | >60 | 40 (50%) |
| Weight, n (%) | <=80 | 27 (50.9%) |
|  | >80 | 26 (49.1%) |
| Height, n (%) | <=170 | 35 (66%) |
|  | >170 | 18 (34%) |
| BMI, n (%) | <=30 | 39 (73.6%) |
|  | >30 | 14 (26.4%) |
| Histological type, n (%) | Epithelioid Cell | 13 (16.2%) |
|  | Spindle Cell | 30 (37.5%) |
|  | Mix | 37 (46.2%) |
| Tumor shape, n (%) | Diffuse | 2 (3.9%) |
|  | Dome | 36 (70.6%) |
|  | Mushroom | 13 (25.5%) |
| OS event, n (%) | Alive | 57 (71.2%) |
|  | Dead | 23 (28.7%) |
| DSS event, n (%) | Alive | 59 (73.8%) |
|  | Dead | 21 (26.2%) |
| PFI event, n (%) | Alive | 50 (62.5%) |
|  | Dead | 30 (37.5%) |
| Age, median (IQR) |  | 61.5 (51, 74.25) |
