# Supplementary material for: N6-Methyladenosine regulator RBM15B acts as an independent prognostic biomarker and its clinical significance in uveal melanoma
Source: Front Immunol. 2022 Aug 8;13:918522. doi: 10.3389/fimmu.2022.918522 (PMC9393712; doi:10.3389/fimmu.2022.918522)
Supplement: Supplementary Table 2 — The descriptions of 20 m6A regulators described in this study. [file Table_2.docx]

**Table S2.** The descriptions of 20 m^6^A regulators described in this study.

| Official symbol | Gene ID | Description | Category |
| --- | --- | --- | --- |
| WTAP | 9589 | WT1 associated protein | Writer |
| VIRMA | 25962 | Vir like m6A methyltransferase associated | Writer |
| METTL3 | 56339 | Methyltransferase like 3 | Writer |
| METTL14 | 57721 | Methyltransferase like 14 | Writer |
| RBM15 | 64783 | RNA binding motif protein 15 | Writer |
| RBM15B | 29890 | RNA binding motif protein 15B | Writer |
| ZC3H13 | 23091 | Zinc finger CCCH-type containing 13 | Writer |
| ALKBH5 | 54890 | AlkB homolog 5, RNA demethylase | Eraser |
| FTO | 79068 | FTO alpha-ketoglutarate dependent dioxygenase | Eraser |
| YTHDC1 | 91746 | YTH domain containing 1 | Reader |
| YTHDC2 | 64848 | YTH domain containing 2 | Reader |
| IGF2BP1 | 10642 | Insulin like growth factor 2 mRNA binding protein 1 | Reader |
| IGF2BP2 | 10644 | Insulin like growth factor 2 mRNA binding protein 2 | Reader |
| IGF2BP3 | 10643 | Insulin like growth factor 2 mRNA binding protein 3 | Reader |
| YTHDF1 | 54915 | YTH N6-methyladenosine RNA binding protein 1 | Reader |
| YTHDF2 | 51441 | YTH N6-methyladenosine RNA binding protein 2 | Reader |
| YTHDF3 | 253943 | YTH N6-methyladenosine RNA binding protein 3 | Reader |
| HNRNPA2B1 | 3181 | Heterogeneous nuclear ribonucleoprotein A2/B1 | Reader |
| HNRNPC | 3183 | Heterogeneous nuclear ribonucleoprotein C | Reader |
| RBMX | 27316 | RNA binding motif protein X-linked | Reader |
