# Supplementary material for: N6-Methyladenosine regulator RBM15B acts as an independent prognostic biomarker and its clinical significance in uveal melanoma
Source: Front Immunol. 2022 Aug 8;13:918522. doi: 10.3389/fimmu.2022.918522 (PMC9393712; doi:10.3389/fimmu.2022.918522)
Supplement: Supplementary Table 3 — online database used in this study. [file Table_3.docx]

**Table S3.** online database used in this study.

| Database | website |
| --- | --- |
| cBioPortal[1, 2] | http://www.cbioportal.org/ |
| CancerMIRNome[3] | http://bioinfo.jialab-ucr.org/CancerMIRNome/ |
| TCGA | https://portal.gdc.cancer.gov/ |
| GeneMANIA[4] | http://genemania.org/ |
| GEPIA2[5] | http://gepia2.cancer-pku.cn/#index |
| UALCAN[6] | http://ualcan.path.uab.edu/ |
| HPA | https://www.proteinatlas.org/ |
| TIMER2.0[7] | http://timer.cistrome.org/ |
| ENCORI[8] | https://starbase.sysu.edu.cn/index.php |
| TISIDB[9] | http://cis.hku.hk/TISIDB/index.php |
| oncomir[10] | http://www.oncomir.org/ |

REFERENCES

1. Gao J, Aksoy BA, Dogrusoz U, Dresdner G, Gross B, Sumer SO, Sun Y, Jacobsen A, Sinha R, Larsson E *et al*: **Integrative analysis of complex cancer genomics and clinical profiles using the cBioPortal**. *Science signaling* 2013, **6**(269):pl1.

2. Cerami E, Gao J, Dogrusoz U, Gross BE, Sumer SO, Aksoy BA, Jacobsen A, Byrne CJ, Heuer ML, Larsson E *et al*: **The cBio cancer genomics portal: an open platform for exploring multidimensional cancer genomics data**. *Cancer Discov* 2012, **2**(5):401-404.

3. Li R, Qu H, Wang S, Chater JM, Wang X, Cui Y, Yu L, Zhou R, Jia Q, Traband R *et al*: **CancerMIRNome: an interactive analysis and visualization database for miRNome profiles of human cancer**. *Nucleic acids research* 2022, **50**(D1):D1139-D1146.

4. Warde-Farley D, Donaldson SL, Comes O, Zuberi K, Badrawi R, Chao P, Franz M, Grouios C, Kazi F, Lopes CT *et al*: **The GeneMANIA prediction server: biological network integration for gene prioritization and predicting gene function**. *Nucleic acids research* 2010, **38**(Web Server issue):W214-220.

5. Tang Z, Kang B, Li C, Chen T, Zhang Z: **GEPIA2: an enhanced web server for large-scale expression profiling and interactive analysis**. *Nucleic acids research* 2019, **47**(W1):W556-W560.

6. Chandrashekar DS, Bashel B, Balasubramanya SAH, Creighton CJ, Ponce-Rodriguez I, Chakravarthi B, Varambally S: **UALCAN: A Portal for Facilitating Tumor Subgroup Gene Expression and Survival Analyses**. *Neoplasia* 2017, **19**(8):649-658.

7. Li T, Fu J, Zeng Z, Cohen D, Li J, Chen Q, Li B, Liu XS: **TIMER2.0 for analysis of tumor-infiltrating immune cells**. *Nucleic acids research* 2020, **48**(W1):W509-W514.

8. Li JH, Liu S, Zhou H, Qu LH, Yang JH: **starBase v2.0: decoding miRNA-ceRNA, miRNA-ncRNA and protein-RNA interaction networks from large-scale CLIP-Seq data**. *Nucleic acids research* 2014, **42**(Database issue):D92-97.

9. Ru B, Wong CN, Tong Y, Zhong JY, Zhong SSW, Wu WC, Chu KC, Wong CY, Lau CY, Chen I *et al*: **TISIDB: an integrated repository portal for tumor-immune system interactions**. *Bioinformatics (Oxford, England)* 2019, **35**(20):4200-4202.

10. Wong NW, Chen Y, Chen S, Wang X: **OncomiR: an online resource for exploring pan-cancer microRNA dysregulation**. *Bioinformatics (Oxford, England)* 2018, **34**(4):713-715.
