# Supplementary material for: N6-Methyladenosine regulator RBM15B acts as an independent prognostic biomarker and its clinical significance in uveal melanoma
Source: Front Immunol. 2022 Aug 8;13:918522. doi: 10.3389/fimmu.2022.918522 (PMC9393712; doi:10.3389/fimmu.2022.918522)
Supplement: Supplementary Table 4 — Univariate and multivariate cox analysis of 20 m6A regulators in uveal melanoma cases. [file Table_4.docx]

**Table S4.** Univariate and multivariate cox analysis of 20 m^6^A regulators in uveal melanoma cases.

| Characteristics | Univariate analysis | |  | Multivariate analysis | |
| --- | --- | --- | --- | --- | --- |
|  | Hazard ratio (95% CI) | P value |  | Hazard ratio (95% CI) | P value |
| RBM15B | 0.031 (0.004-0.229) | **<0.001** |  | 0.053 (0.006-0.426) | **0.006** |
| VIRMA | 2.504 (0.985-6.365) | 0.054 |  | 0.387 (0.020-7.540) | 0.531 |
| IGF2BP2 | 0.110 (0.030-0.404) | **<0.001** |  | 0.194 (0.035-1.057) | 0.058 |
| HNRNPA2B1 | 1.207 (0.514-2.835) | 0.666 |  |  |  |
| IGF2BP1 | 2.459 (1.046-5.780) | **0.039** |  | 0.612 (0.129-2.911) | 0.537 |
| YTHDF3 | 2.864 (1.126-7.282) | **0.027** |  | 4.552 (0.272-76.064) | 0.291 |
| IGF2BP3 | 3.658 (1.434-9.329) | **0.007** |  | 1.980 (0.601-6.525) | 0.261 |
| HNRNPC | 1.804 (0.763-4.268) | 0.179 |  |  |  |
| RBM15 | 1.143 (0.491-2.658) | 0.757 |  |  |  |
| RBMX | 0.601 (0.263-1.377) | 0.229 |  |  |  |
| METTL14 | 1.138 (0.485-2.670) | 0.766 |  |  |  |
| YTHDC2 | 1.162 (0.495-2.723) | 0.730 |  |  |  |
| METTL3 | 1.315 (0.549-3.151) | 0.539 |  |  |  |
| ZC3H13 | 0.875 (0.382-2.005) | 0.752 |  |  |  |
| WTAP | 1.321 (0.567-3.079) | 0.519 |  |  |  |
| YTHDF1 | 3.225 (1.193-8.719) | **0.021** |  | 0.940 (0.239-3.689) | 0.929 |
| YTHDC1 | 1.347 (0.563-3.221) | 0.503 |  |  |  |
| FTO | 1.053 (0.464-2.392) | 0.901 |  |  |  |
| YTHDF2 | 1.580 (0.668-3.739) | 0.298 |  |  |  |
| ALKBH5 | 2.044 (0.870-4.800) | 0.101 |  |  |  |
