# Supplementary material for: N6-Methyladenosine regulator RBM15B acts as an independent prognostic biomarker and its clinical significance in uveal melanoma
Source: Front Immunol. 2022 Aug 8;13:918522. doi: 10.3389/fimmu.2022.918522 (PMC9393712; doi:10.3389/fimmu.2022.918522)
Supplement: Supplementary Table 5 — Logistic regression analysis of the correlation between RBM15B expression and clinical characteristics. [file Table_5.docx]

**Table S5.** Logistic regression analysis of the correlation between RBM15B expression and clinical characteristics.

| Characteristics | Total(N) | Odds Ratio(OR) | P value |
| --- | --- | --- | --- |
| Age (>60 vs. <=60) | 80 | 0.669 (0.275-1.611) | 0.372 |
| Gender (Male vs. Female) | 80 | 0.903 (0.371-2.191) | 0.822 |
| Pathologic stage (Stage III&Stage IV vs. Stage II) | 79 | 0.514 (0.207-1.249) | 0.145 |
| Clinical stage (Stage III&Stage IV vs. Stage II) | 80 | 0.356 (0.140-0.873) | **0.026** |
| Tumor shape (Dome&Mushroom vs. Diffuse) | 51 | 68362834.257 (0.000-NA) | 0.996 |
| Histological type (Spindle Cell&Mix vs. Epithelioid Cell) | 80 | 4.111 (1.140-19.573) | **0.044** |
